# Forging a Frailty-Ready Healthcare System to Meet Population Ageing

**DOI:** 10.3390/ijerph14121448

**Published:** 2017-11-24

**Authors:** Wee Shiong Lim, Sweet Fun Wong, Ian Leong, Philip Choo, Weng Sun Pang

**Affiliations:** 1 Institute of Geriatrics & Active Ageing, Tock Seng Hospital, Singapore 308433, Singapore; Ian_Leong@ttsh.com.sg; 2 Khoo Teck Puat Hospital, Singapore 768828, Singapore; wong.sweet.fun@ktph.com.sg (S.F.W.); pang.weng.sun@ktph.com.sg (W.S.P.); 3 National Healthcare Group, Singapore 138543, Singapore; philip_choo@nhg.com.sg; 4 Geriatric Education & Research Institute, Singapore 768024, Singapore

**Keywords:** population ageing, frailty, healthcare delivery, integrated care, end-of-life care

## Abstract

The beginning of the 21st century has seen health systems worldwide struggling to deliver quality healthcare amidst challenges posed by ageing populations. The increasing prevalence of frailty with older age and accompanying complexities in physical, cognitive, social and psychological dimensions renders the present *modus operandi* of fragmented, facility-centric, doctor-based, and illness-centered care delivery as clearly unsustainable. In line with the public health framework for action in the World Health Organization’s World Health and Ageing Report, meeting these challenges will require a systemic reform of healthcare delivery that is integrated, patient-centric, team-based, and health-centered. These reforms can be achieved through building partnerships and relationships that engage, empower, and activate patients and their support systems. To meet the challenges of population ageing, Singapore has reorganised its public healthcare into regional healthcare systems (RHSs) aimed at improving population health and the experience of care, and reducing costs. This paper will describe initiatives within the RHS frameworks of the National Health Group (NHG) and the Alexandra Health System (AHS) to forge a frailty-ready healthcare system across the spectrum, which includes the well healthy (“living well”), the well unhealthy (“living with illness”), the unwell unhealthy (“living with frailty”), and the end-of-life (EoL) (“dying well”). For instance, the AHS has adopted a community-centered population health management strategy in older housing estates such as Yishun to build a geographically-based care ecosystem to support the self-management of chronic disease through projects such as “wellness kampungs” and “share-a-pot”. A joint initiative by the Lien Foundation and Khoo Teck Puat Hospital aims to launch dementia-friendly communities across the island by building a network comprising community partners, businesses, and members of the public. At the National Healthcare Group, innovative projects to address the needs of the frail elderly have been developed in the areas of: (a) admission avoidance through joint initiatives with long-term care facilities, nurse-led geriatric assessment at the emergency department and geriatric assessment clinics; (b) inpatient care, such as the Framework for Inpatient care of the Frail Elderly, orthogeriatric services, and geriatric surgical services; and (c) discharge to care, involving community transitional care teams and the development of community infrastructure for post-discharge support; and an appropriate transition to EoL care. In the area of EoL care, the National Strategy for Palliative Care has been developed to build an integrated system to: provide care for frail elderly with advance illnesses, develop advance care programmes that respect patients’ choices, and equip healthcare professionals to cope with the challenges of EoL care.

## 1. Introduction

The beginning of the 21st century has seen an exponential growth in population ageing. According to the United Nations (2017) report on World Population Prospects, there is an estimated 962 million people aged 60 years and above, who comprise 13 percent of the global population [1]. With a growth rate of about three percent per year, this figure is projected to hit 1.4 billion in 2030. Globally, the oldest old population, which is variously defined as 80 or 85 years and older, has emerged as the fastest growing age segment, especially in developed countries [2].

Although populations around the world are rapidly ageing, evidence that increasing longevity is accompanied by an extended period of good health is scarce [3]. In most populations, the increase in life expectancy outstrips the increase in healthy life expectancy; as a consequence, a greater proportion of one’s lifespan is spent with disability. This constitutes an expansion of morbidity, which is in contrast to the Fries (1980) concept of the compression of morbidity [4]. The epidemiological transition away from communicable, maternal, infant, and nutritional disease is also offset by an increase in non-communicable chronic disease, along with increased disease burden from multi-morbidity and geriatric syndromes such as frailty and dementia [5]. Clearly, disease-based conceptualisations are inadequate proxies for health in an older person. Rather than the presence or absence of disease, the most important consideration for an older person is likely to be their functional ability. This conceptual shift was reflected in the World Health and Ageing Report of the World Health Organization (WHO), which emphasised function as an important outcome for ageing populations by highlighting the concept of raising intrinsic capacity throughout the life course [5,6]. 

The complementary perspective is the prevention of frailty, which has physical, cognitive, social, and psychological dimensions [7]. Frailty is a geriatric syndrome characterised by the loss of physiologic reserves, which increases the vulnerability of the older adult following trivial stressor events, and leads to a higher risk of negative health-related outcomes [8]. The prevalence of frailty in community-dwelling older adults in the Asia-Pacific region is approximately 3.5–27% [9]. The “frailty syndrome” has been described as an emerging public health priority, as it may represent a vicious cycle responsible for the onset of negative health-related outcomes, a transition phase between successful ageing and disability, and a condition to target for restoring robustness in the individual at risk [10]. The body of evidence indicates that a large proportion of community-dwelling older people present risk factors for major health-related events and unmet clinical needs [11]. If left unaddressed, this may result in increased disability and the increased consumption of health and social care resources; in one study, the incremental effect on ambulatory health expenditure approximates an additional €1500 per frail person per year [12]. It can also result in significant caregiver burden and the accompanying informal costs of caregiving, particularly in Asian populations, which are often heavily influenced by notions of filial piety and obligatory care [13,14]. 

Against this backdrop, health systems worldwide are struggling to deliver quality healthcare amidst challenges posed by ageing populations, the complexity of health states in old age, and increasingly complex technology, which all have contributed to escalating costs. Most healthcare systems in the world have been built on the disease-based acute care model, which originated in the clinical service model for handling acute and defined disease episodes, and is singularly inadequate to meet the challenges ushered by the new era of multiple interacting chronic diseases and the accompanying complexity of the physical, cognitive, social, and psychological dimensions of the frailty syndrome [10,15]. This provides the impetus for the recent discourse surrounding the utility of the frailty concept in guiding the development of health policies in caring for older people. Woo (2017) eloquently argued that health systems and models of care should be realigned and redesigned to be fit for purpose and better address the unmet needs of frail older people [16]. 

The public health framework for healthy ageing promoted by the WHO calls for four key areas of action by governments on healthy ageing: aligning health systems to the needs of the older populations they now serve; developing systems to provide long-term care; ensuring everyone can grow old in an age-friendly environment; and improving measurement, monitoring, and understanding [5,6]. In line with the public health framework for action in the WHO’s World Health and Ageing Report, meeting these challenges will require a systemic reform of healthcare delivery that is integrated, patient-centric, team-based, and health-centred through building partnerships and relationships that engage, empower, and activate both the patients and their support systems. This paper will mainly focus on the first and third areas of the WHO report, using Singapore as a case study.

## 2. Singapore as a Case Study 

Singapore has a multiethnic population of about 5.7 million. The low infant mortality (2.4 per 1000 live births) and high life expectancy (82.9 years) attests to the standard of healthcare [17]. Since the mid-1960s, when the country developed into an economic powerhouse, Singapore’s population began graying at a speed that matched many other industrialised economies. By 2000, with at least 7% of its population aged 65 and above, Singapore had become an ageing society. In 2016, 13.7% of the population was aged 65 and above, and Singapore is forecast to breach the 14% mark and become an aged society soon [17]. Not surprisingly, population ageing has been identified as one of the major challenges confronting the healthcare system [18].

Singapore’s healthcare delivery system is a dual one of public and private care: 80% of inpatient care is provided in public hospitals, while 80% of primary health care is provided by independently employed family physicians [19]. A principal feature of the healthcare philosophy for the public system is that of individual responsibility for health and the need for copayment for services provided. Public healthcare facilities are primarily designed to provide subsidised healthcare services to Singaporeans, and consist of hospitals for inpatient services and numerous polyclinics offering outpatient services. Although wholly owned by the government, the public sector hospitals are operated as autonomous organisations in order to instill financial discipline and devolve operational autonomy [19]. 

The traditional care delivery system tends to be facility based, hospital-centered, and more siloed and fragmented in terms of care coordination [20]. While the advent of chronic disease management in the early 2000s has facilitated the follow through and coordination of care processes across the lifetime of an illness [19], this approach is inadequate in the face of confluent multi-morbidity and ill-defined geriatric syndromes that do not fit the single-disease model [5,10]. Evidence-based clinical guidelines work best in discrete conditions, but have not for the most part, focussed on the integration of multiple interacting and possibly competing chronic conditions within individuals [21]. Similar to the worldwide experience, acute hospitals and emergency departments were generally neither elder-friendly nor frailty-ready. Not surprisingly, hospitalised frail older adults constitute an at-risk population that was vulnerable to adverse post-hospitalisation outcomes such as functional disability, institutionalisation, and mortality [22]. Post-discharge community services were underdeveloped to support the successful transition from discharge to home, resulting in recurrent hospitalisations accruing from an index admission. The lack of a systematic framework for advance care planning meant that the perspectives and preferences of patients regarding their health and treatment choices, which are especially pertinent in the context of end-of-life (EoL) care, were often not incorporated into the care plan. Finally, at the health policy level, the funding mechanism in Singapore was previously based upon episodes of care, and did not provide incentives for public healthcare providers to efficiently organise and coordinate care across the whole range of services, or develop preventative and health promotion activities [19].

### 2.1. Concept of Regional Healthcare Systems (RHS)

To meet healthcare challenges such as population ageing, increased chronic disease burden, and the need to manage future growth in healthcare manpower and spending, the healthcare 2020 master plan was announced in 2012 to improve the accessibility, affordability, and quality of healthcare in Singapore [23]. One strategy that is being adopted to better integrate care across different settings is re-organising the healthcare system into regional health systems (RHSs). Each RHS comprises an acute general hospital working closely with community hospitals, nursing homes, hospices, home care, and day rehabilitation providers, as well as government polyclinics and private general practitioners (GPs) within the geographical region. The purpose of the RHS is to foster the vertical integration of services, and enhance synergy and economies of scale to improve the quality of healthcare while keeping medical costs affordable. From the patient perspective, the provision of integrated, seamless, and holistic care by the RHS enables patients and their caregivers to navigate across providers more easily. It also empowers them to manage their care needs across different stages of their healthcare journey, from diagnosis and treatment through to post-discharge follow-up. 

Public healthcare facilities were initially divided into six RHSs. Preliminary results indicate that integration efforts to enhance the primary, intermediate, long-term, and home care sectors, as well as consolidate networks between hospitals and these care providers, have helped to streamline processes, support the faster recovery of patients, and shorten the length of hospitalisation [24]. To better meet Singaporeans’ future healthcare needs, the Ministry of Health recently announced that the healthcare system will be further re-organised into three integrated clusters from the existing six RHSs [25]. In the central region, the National Healthcare Group (NHG) and the Alexandra Health System (AHS) will form one cluster under the National Healthcare Group. In the eastern region, the second cluster under SingHealth will comprise Singapore Health Services (SingHealth) and the Eastern Health Alliance, whilst the National University Health System (NUHS) will merge with Jurong Health Services to form the third cluster under NUHS in the western region. This reorganisation provides an opportunity to further foster integration through the design and coordination of services within the cluster, and also inter-cluster cooperation for innovations in care delivery. The healthcare funding structure will also be aligned to place a greater focus on health prevention and maintenance programmes that incentivise individuals and families to stay healthy and be active participants in their health matters.

### 2.2. Blueprint of the RHS Framework for the Central Region

#### 2.2.1. Challenges and Key Directions

The central region serves around two-fifths of Singapore’s population. Since many of the older housing estates are located in the central region, this catchment area also serves a higher proportion of the elderly population. Based upon results of the 2015 census in the central region, more than two-thirds of older adults aged 60 years and above are either living with illness (57.6%) or frailty (14.1%) [20]. Frailty was defined empirically from database-derived variables using the phenotypic approach. The typical profile of the frail group is older age, female gender, lower socio-economic status, living alone or with limited family support, physical disability, and increased care needs. The top contributors to the frailty indicators are stroke and dementia. 

**Figure 1:**
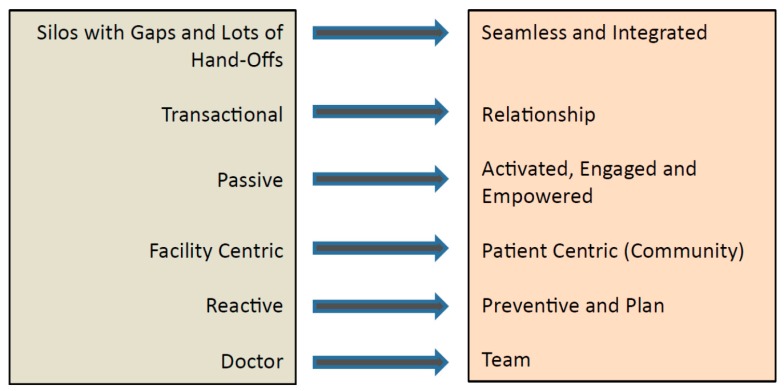
Paradigm shift in healthcare delivery.

Against this backdrop, it is clear that the present modus operandi of fragmented, siloed, and facility-centric healthcare with lots of hand-offs and care delivery organised around the doctor is both untenable and unsustainable. The patient is often a passive recipient of care, with care provision often occurring on a transactional basis in reaction to a medical need or a crisis presentation. Social determinants of health are not adequately addressed during acute care episodes. There is also a big discrepancy in the quality of care between hospital and home, with little community involvement. The reorganisation of the healthcare system into RHSs prompted a paradigm shift in the approach toward ageing and health, namely: to move beyond the hospital to the community; to move beyond quality to value; and to move beyond healthcare to health [23]. In the central region, this translates into the reorganisation of care to achieve seamless and integrated care across the continuum of health that emphasises prevention and planning, and actively engages community partners through a team-based approach (Figure 1). Importantly, effective engagement with patients and their caregivers is not about achieving patient “compliance” with professional recommendations, but rather about promoting dialogue and building trusting relationships to activate patients, families, and their caregivers, so that they are activated, engaged, and empowered in the care process. There is a growing body of evidence showing that patients who are more activated have better health outcomes and care experiences [26].

#### 2.2.2. Blueprint of the RHS Framework 

**Figure 2:**
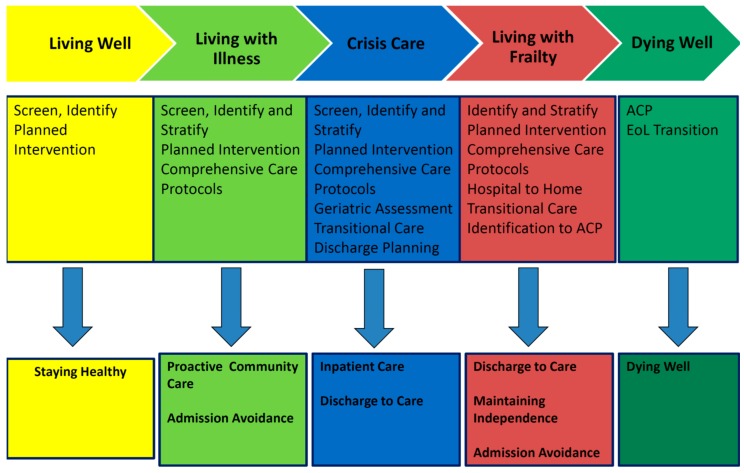
The National Health Group (NHG) blueprint across the care continuum. EoL: end-of-life.

A holistic framework is needed to provide a clear blueprint for the systemic shift towards forging a frailty-ready healthcare system that spans the care continuum, including the robust (“living well”), the healthy with chronic diseases (“living with illness”), those who become acutely unwell or develop complications from chronic diseases (crisis and complex care), the frail who are vulnerable to adverse outcomes (“living with frailty”), and finally, the terminally ill (“dying well”) [20]. Depending on the care needs, a calibrated modular bundle of care services is then delivered via multi-disciplinary teams. These cover the domains of staying healthy, proactive community care, admission avoidance, inpatient care, discharge to care, maintaining independence, and dying well (Figure 2). For instance, for the “living with frailty” group, the relevant bundle of care services would include discharge to care, maintaining independence, and admission avoidance. 

### 2.3. Community Initiatives for Ageing-in-Place

Over the next two decades, the rapid demographic shift in Singapore will manifest in population ageing, lower labour growth, and shrinking family sizes. With an increasing number of seniors and weaker family support, the demand for aged care facilities and institutionalisation will grow. Yet, ageing-in-place and at home remain the preference of many local seniors [27]. In line with the WHO’s priority areas of aligning health systems to the needs of the older populations they serve and ensuring everyone can grow old in an age-friendly environment [6], one key thrust of the RHS strategy is to orient systems around intrinsic capacity by developing community initiatives that ensure access to older person-centred services that support ageing-in-place. It is important to address the attendant social factors that can influence health choices and behaviours, and build trusting relationships between healthcare workers and patients in their homes and the community where they make their health choices [28]. This is especially salient in the central region, where the majority of the older population comes from the lower socio-economic strata and resides in older public housing estates. We describe two examples to illustrate how programmes premised on a community-centric population health approach can help meet the healthcare and social needs of the frail elderly to support ageing-in-place.

#### 2.3.1. Wellness Kampungs

To build resilience and sustainability into tomorrow’s health landscape, the Alexandra Health System adopted the approach of going upstream to address social determinants of health by creating supported self-managed communities. This led to the development of three community wellness centres in Yishun in the northern part of Singapore. To convey the overall aspirational goal of achieving healthy, active, and engaged residents, the name “wellness kampung” (yǎng shēng cūn) was chosen. This name combines elements from three major spoken languages in Singapore, and the word “kampong” connotes inclusiveness, and the “gotong royong” spirit—a Malay expression meaning the communal helping of one another. Based on the concepts of the Ibasho Café [29], the wellness kampungs harnessed good design concepts of open central spaces to facilitate the congregation and social interaction of people together in a common activity, thus fostering a strong sense of community bonding between the residents to epitomise the “kampung spirit”. Since the start of operations in April 2016, the three wellness kampungs have served more than 1500 residents through their various healthy lifestyle programmes (daily work-outs, cooking demonstrations, and recipe sharing), social engagement activities (computing, conversational English, and calligraphy), and health-related activities (health screening, literacy, and intervention programmes). 

Bridging the health and social divide in the programming of the activities at the wellness kampung was intentional. For instance, “Share-a-Pot” is a community-based project that was developed to improve the nutrition of community-dwelling seniors [30]. It is founded on the principles of good nutrition, working hand-in-hand with physical activity in a social environment to “build bones, brawn (muscle), brain (cognitive reserve), and bonds (social engagement and reciprocity)”. These activities increase social networking and community participation, enhance the sense of belonging and trust, and develop reciprocity between neighbors. The enhanced sense of social capital in turn strengthens the community’s resilience and contributes towards building a geographically-based care ecosystem that redefines the communal care experience and facilitates the organic growth of local communities to live healthy lifestyles; supports the self-management of chronic disease; and maintains fitness and independent function. Each Wellness Kampung is supported by a responsive healthcare team that is embedded and easily accessible.

#### 2.3.2. Dementia Friendly Communities

The Lien Foundation and Khoo Teck Puat Hospital (KTPH) developed the “Forget Us Not” initiative, which aims to launch dementia-friendly communities (DFC) across the island by building a network comprising community partners, businesses, and members of the public [31]. A DFC is a neighbourhood where residents, businesses and services, and the community at large are aware of dementia and understand how to better support persons with dementia (PWDs) and their caregivers. A DFC also provides a secure environment in which PWDs can move around safely, thus reducing the stress of caregivers of PWDs by helping to look out for their loved ones. Through multi-stakeholder collaborations to optimise strategies and participation, DFCs emphasise an age-friendly environment for PWDs in the community, by the community, and for the community, with the collaborative effort from the government, private sector, non-governmental organisations, and members of the public [32].

Since the first DFC was piloted in Chong Pang in 2015, the ground-up movement has quickly gained momentum, with the launch of further DFCs in other housing estates throughout Singapore. In each DFC, trained citizens-on-patrol, grassroots leaders, volunteers, students, and the staff of business entities function as lookouts to assist PWDs in the community. Training is provided in the following areas: features of a DFC, common signs and symptoms of dementia, how to reach out to PWDs in the community, and how to communicate with PWDs [31]. To date, this initiative has trained about 17,000 people and worked with about 70 organisations to raise awareness of dementia [33]. In addition, each DFC features a safe return system comprising a network of four to five “Go-To Points” where PWDs who are lost can be taken by members of the public. The go-to points also serve as community resource centres for caregivers to get information about dementia, attend classes, and be linked with relevant services.

### 2.4. Admission Avoidance

Based on the 2015 census, the proportion of patients requiring crisis care at their first NHG visit increased exponentially with age, from 12.7% in the 60–64 age group and 27.1% in the 75–79 age group to 52.6% in the 85+ age group [20]. Coupled with the fact that older patients often have longer lengths of stay, not surprisingly, this concomitant surge in demand for hospitalisation has resulted in a bed crunch situation in the public sector hospitals in recent years [34]. This provided the impetus for a comprehensive multi-pronged strategy for admission avoidance that spans community outreach programmes for those “living with illness” through to the “living with frailty” and “dying well” groups (Figure 2). For the former, the goal is to tap into community networks with support from an embedded healthcare team to help the senior residents remain well in their own homes and communities, with access to rapid support that is close to home in times of augmented health needs, and thus reduce the need for presentation to the emergency department and hospitalisation. For the latter two groups, this involves the development of models of care to address the needs of the frail elderly, such as joint initiatives with long-term care facilities; nurse-led geriatric assessment at the emergency department (ED); geriatric assessment clinics; and home-based palliative care services. 

#### 2.4.1. Project Care

Since 2009, Project Care has been an ongoing collaboration between Tan Tock Seng Hospital (TTSH) and affiliated nursing homes in central Singapore [35]. It aims to reduce unnecessary admissions to hospitals through the identification of residents with poor prognosis, conversations about advance care planning with these patients and their family members, care coordination for nursing home residents, and the upskilling of nursing home staff in managing common end-of-life symptoms. Currently, more than 1500 nursing home residents have completed advance care plan discussions. For residents with advanced dementia or other terminal conditions who choose to spend their last days in the nursing home, care is then delivered through the collaborative efforts of both the TTSH and nursing home teams. This has helped residents recover from acute reversible conditions or pass away in comfort in a familiar and conducive environment under the care of nurses who understand them well, resulting in higher family satisfaction. 

#### 2.4.2. Emergency Department (ED) Geriatric Screening and Intervention 

The ED at TTSH runs an extremely busy service with an annual attendance of 160,000 patients. To meet the challenges posed by the variegated group of elderly patients with differing risk profiles [36], an innovative nurse-led model of care was developed to risk stratify all patients aged 65 years and older presenting to the ED, followed by rapid geriatric screening and intervention for at-risk seniors. Using the triage risk screening tool to risk-stratify, the geriatric emergency medicine nurse performs a focussed geriatric screening lasting 15–30 min to at-risk seniors with a triage risk screening tool score of 2 or more who were planned for discharge [37]. Interventions include the timely management of identified clinical issues, and where necessary, referrals to the physiotherapist and occupational therapist; the geriatric assessment clinic; post-acute care at home services; and community support services. Upon discharge, advice regarding fluid management, falls prevention, sleep hygiene, and active lifestyles were provided where necessary. The most common positive findings from nurse screenings were fall risk (65.0%), vision (61.4%), and footwear (58.2%) issues. More than a quarter (28.2%) of patients were referred to a geriatric clinic. Compared with controls, the intervention group had significant preservation of basic and instrumental activities of daily living at 12 months [38]. There was also a trend towards reduction in ED reattendance (odds ratio (OR): 0.75, confidence interval (CI) 0.55–1.03, *p* = 0.07) and hospitalisation (OR: 0.77, CI 0.57–1.04, *p* = 0.09) [38].

#### 2.4.3. Geriatric Assessment Clinics

The frail elderly patients often present with health states in older age that are not captured by traditional disease classifications, which are commonly known as geriatric syndromes. In addition, there are often attendant functional, psychological, and social issues. Thus, specialised geriatric assessment clinics are available to provide comprehensive geriatric assessments in order to identify and manage geriatric syndromes, sensory impairment, functional disability, and psychosocial issues, as part of the secondary prevention efforts to avoid admissions in the at-risk frail elderly [39]. For instance, at TTSH, the clinic is helmed by a nurse clinician, who first performs a comprehensive geriatric assessment based upon a standardised protocol, before evaluation by the doctor. Depending on identified needs, the patient is then seen by other members of the multi-disciplinary team such as physiotherapists, occupational and speech therapists, pharmacists, dietitians, or social workers. The clinic receives referrals from the ED, primary care, and hospital doctors for the evaluation of geriatric syndromes and related issues in older persons aged 65 years and above. Tie-ups with the ED and primary care polyclinics allow early access to the geriatric assessment clinic. 

### 2.5. Inpatient Care

The frail elderly are an at-risk group with complex interacting comorbidities, polypharmacy, and attendant functional and psychosocial issues, who are vulnerable to adverse outcomes such as iatrogenesis, functional disability, institutionalisation, and mortality as a result of hospitalisation [8]. The development of innovative models of care to support responsive frailty-ready acute care services for older people thus remains a high-priority focus. 

#### 2.5.1. Framework for Inpatient Care of the Frail Elderly 

**Figure 3:**
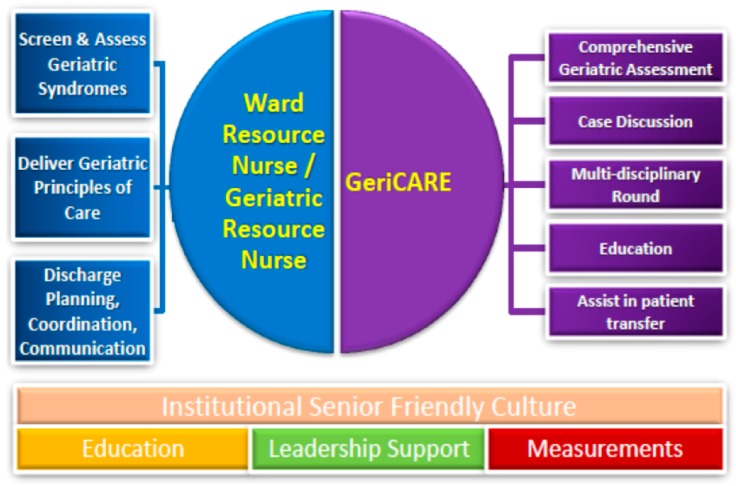
Overview of the Framework for Inpatient care of the Frail Elderly (FIFE) model (Reproduced with permission from Associate Professor Tan Thai Lian).

The prevalent model of care is to cohort frail older persons in acute care of the elderly wards that aim to provide good-quality older person-centred care in accordance with the principles of good geriatric care [40]. This model is increasingly inadequate to meet the burgeoning demand of care needs imposed by population ageing, and the concomitant increase in the number of frail patients with complex comorbidities who would still require specialty-related treatment. Against this backdrop, the Framework for Inpatient care of the Frail Elderly (FIFE) was formulated in 2014 to design a system of care that reaches out to frail older patients in non-geriatrics specialty wards (Figure 3). The principal objective of the framework is to promulgate geriatric principles of care throughout the hospital system to render it truly senior-friendly and frailty-ready. There are two core components in this framework: the Nurses Improving Care for Healthsystem Elders (NICHE) arm is helmed by the geriatric resource nurses and the local champions in each ward, the ward resource nurses [41]. They work closely with the nursing staff in the ward to provide four key elements of care through an interprofessional, team-based, patient-centred care approach: (1) screening and flagging of at-risk elderly patients who may benefit from comprehensive geriatric assessment; (2) early discharge planning; (3) point of contact to serve as a liaison between family/caregivers and the care team; and (4) implementing evidence-based geriatric principles of care systematically in the ward, such as components of the Hospital Elder Life Programme in delirium prevention [42]. The second arm of FIFE is the professional arm (GeriCARE), which is helmed by advanced practice nurses paired with a geriatric nurse assessor, and supported by a geriatric medicine physician. Together, they form the mobile geriatrics team, which provides comprehensive geriatric assessments and recommendations for intervention in the screen-positive population, and also functions as an expert resource for the ward resource nurses and geriatric resource nurses. 

#### 2.5.2. Orthogeriatric Service 

A five-year programme modelled after Geriatric Fracture Centres (GFC) was set up in TTSH in 2011 to improve the quality of hip fracture care for older persons. The key strategy for GFCs is comanaged care defined by interdisciplinary involvement and the integration of orthopedic surgeons, geriatricians, anesthetists, rehabilitation physicians, nurses, physiotherapists, occupational therapists, care managers, social workers, and dieticians working together with shared ownership and equal responsibilities [43]. Building upon the GFC model, the strategies adopted include prompt admission from the emergency department to orthopedic wards, comanagement between orthopedics and geriatric medicine with interdisciplinary team involvement, and standardised care bundles (care path), together with patient and family education using a “hip fracture booklet”. Key measures were instituted, namely: (1) “Fitness for Op Criteria” to expedite surgery within 48 h of admission; (2) extending rehabilitation beyond discharge; and (3) interdisciplinary hip fracture clinic to standardise care and improve osteoporosis treatment and falls assessment and prevention. As a result of these measures, the time to surgery within 48 h was increased to 77% from a baseline of 35%. The 30-day admission rate was 1.3%, hip fracture inpatient mortality rate was 1.4%, and the one-year mortality rate was 12.1% [44]. These good postoperative results are comparable to GFCs worldwide, and support comanaged interdisciplinary care involving the geriatrician as the standard of good elderly hip fracture care to improve outcomes for elderly patients with hip fractures.

#### 2.5.3. Geriatric Surgical Services

The Geriatric Surgery Service of KTPH started in 2007 to cater to the complex and multifaceted needs of elderly patients who are undergoing surgery. A major milestone was the incorporation of the transdisciplinary model of care since 2009, which is underpinned by an ethos of flattening organisational hierarchy to enhance team communication, promote patient-centricity, and facilitate the role enhancement of team members. Programme evaluations using the cumulative sum curve methodology demonstrated a sustained pattern of good outcomes, which were measured mainly by functional recovery after major surgery [45]. In 2012, the recognition that frailty predisposed patients to major complications after surgery led to the development of new processes, including prehabilitation [46]. Funded by the Healthcare Quality Improvement Fund, this community-based programme, termed “Start-to-Finish”, delivers comprehensive care across the continuum from diagnosis, prehabilitation, and surgical management, through to functional recovery and social integration. After the successful pilot in the colorectal surgery department, the programme has since been extended to other departments of abdominal surgery. This novel approach of transinstitutional transdisciplinary care has demonstrated good surgical outcomes, including medium-term functional outcomes. The mean length of stay decreased from 11.0 days to 8.4 days (*p* = 0.029), and all elective patients who received prehabilitation achieved full functional recovery at six weeks [47]. In addition, the rates of major complication and mortality were reduced from 30.8% to 5.3% and from 9.6% to 1.7%, respectively [48]. 

### 2.6. Discharge to Care

#### 2.6.1. Hospital-to-Home

Good discharge planning and post-discharge support are necessary to ensure a smooth transition from hospital to home. Transitional care teams foster care by multi-disciplinary teams to support patients in their homes initially after discharge, and ensure that caregivers are able to provide proper care for the patients. The development of community care infrastructure to provide post-discharge care is also integral to support care transformation efforts to extend care beyond hospitals to the community.

##### Ageing-in-Place Community Care Teams (AIP-CCT)

The Alexandra Health System implemented a comprehensive ageing-in-place programme to cater to the frail elderly who have more ED visits and higher utilisation of hospital services. The goal is to reduce avoidable hospital admissions and improve the quality of life of older people and their caregivers. The community nurse conducts home visits to review their care needs, develop and negotiate a comprehensive care plan, and coordinate follow-up care [49]. Depending on the needs of the patient, follow-up visits might be conducted by a nurse and/or other members of the geographical-based community care team (CCT), such as physiotherapists, occupational and speech therapists, pharmacists, dietitians, social workers, or other community partners. This ensures the continuity of education and care efforts within the home environment, for instance: teaching how to use a blood glucose monitoring kit by the community nurse; teaching simple strengthening exercises to foster independence by the physiotherapist; and the review of medication compliance by the pharmacist. The frequency of visits depends on a person’s needs. Medical inputs to care are provided by a part-time geriatrician or the patient’s primary doctor. Since its implementation, the ageing-in-place community care team (AIP-CCT) has been successful in optimising the use of hospital resources and reducing hospital admissions by 67% [6].

##### Transitional Care (TC) Service

To support care transitions, three care coordination initiatives have been started in TTSH since 2008. The Aged Care TransitION (ACTION) initiative aims to coordinate post-discharge care through inpatient discharge planning and service-matching to appropriate service providers, with the intent of easing the transition of elderly patients with complex care issues back to the community and reducing readmission rates. The Virtual Hospital initiative targets frequent admitters (i.e., patients with three or more admissions in a year) through post-discharge monitoring and collaborations with primary care and community care providers to reduce unnecessary hospital bed days and emergency attendances. Lastly, the Post-Acute Care at Home initiative provides post-discharge supportive care in the following areas: stabilise and rehabilitate patients with sub-acute phases of illness at their home; provide appropriate home care support to promote better self-care; and promote caregiver competence in managing homebound patients to reduce the need for institutionalised care. 

The ACTION, Virtual Hospital, and Post-Acute Care at Home programmes were consolidated into Transitional Care (TC) Service since July 2016 for a single point of contact to coordinate the care plans of complex patients and support their safe, coordinated, and timely transition from hospital to the community and home. The target group includes patients with frequent ED attendance for medical conditions that can be potentially managed at home, and those with multiple chronic conditions, limited social support, and who require the close monitoring of their medical conditions. With support from a multi-disciplinary team of doctors, nurses, allied health professionals, and administrators, the TC Service provides post-discharge follow-up through telephonic reviews, home visits, and the coordination of care with a network of community and primary care partners. Since its implementation on 4 July 2016 to 31 March 2017, there was an 18.2% reduction in ED attendances, and a 62.5% reduction in avoidable admissions.

##### Whampoa Community for Successful Ageing (ComSA)

Run by the Tsao Foundation, the Community for Successful Ageing (ComSA) center is housed in the Whampoa Community Centre. It aims to catalyse the development of a “community of care” in the Whampoa district in the central region [50] by offering a comprehensive range of primary care, geriatric services, case management, preventive care, and wellness programmes that support the ageing-in-place of the residents under one roof. In partnership with the NHG, ComSA jointly provides transitional care for frail elderly patients who have been discharged from hospital to home. This comprises visits to the primary care clinic at ComSA or referrals to case managers for psychosocial support or other services around Whampoa. To cater to the needs of those who are frailer, ComSA also provides daycare services or home visits for their healthcare needs. 

#### 2.6.2. Appropriate Transition to End-of-Life Care 

One of the recommendations of the Report on the National Strategy for Palliative Care (2011) was that palliative care should be delivered in a coordinated manner that ensures continuity of care across settings and over time [51]. The reorganisation of delivery of palliative care based on the regional health system model afforded the opportunity to develop and strengthen effective networks of collaboration between public, private, and voluntary welfare organisation sector providers to facilitate access to seamless and holistic care during the transition to end-of-life care. Strategies to enhance care integration include maximising the use of platforms or means for collaboration and communication between service providers, including the use of information technology, as well as encouraging the involvement of primary care physicians in the provision of palliative care at home and in nursing homes. 

Currently, there is a comprehensive range of palliative care services in acute hospitals (KTPH and TTSH), community hospitals, inpatient hospices, homecare (home hospice and home medical services), daycare (day hospice), and nursing homes. In line with the recommendations of the National Guidelines for Palliative Care released in 2014 [52], a primary provider is identified to coordinate care. To ensure continuity of care during the transition between different care settings, there is a handover of necessary information to the receiving service provider. Where appropriate, referrals are made to other service providers for care needs that fall beyond the usual scope of service, such as personal care. Moving forward, it is important to train and equip healthcare professionals and caregivers to cope with the challenges of EoL care. 

Another key component of the strategy is raising public awareness of advance care planning (ACP). By providing the opportunity for conversations that enable patients to make choices about future personal plans, ACP helps to ensure that patients’ wishes are respected in the event that they become incapable of participating in treatment decisions, and allows for EoL treatment to be consistent with patient preference [53,54]. In concert with the Agency for Integrated Care, a comprehensive strategy was developed to facilitate an ongoing communication process that happens across the life stages, ranging from general ACP through to disease-specific ACP for chronic diseases with complications and organ failure, and finally the preferred plan of care for patients with advanced illness. Informative educational and publicity materials for healthcare staff and patients are available at the Living Matters resource site [55]. Multi-agency collaborative community engagement projects involving religious organisations, grassroots agencies, the Ministry of Health, and the arts community help to promote the awareness and acceptance of ACP. For instance, “Die Die Must Say” (sǐ dōu yào jiǎng) is a grassroots campaign that uses getai (literally, song stage) life-stage performances during the Ghost Festival to engage the elderly Chinese Singaporeans in “die-logues” or conversations about death and dying, thus helping to de-mystify and de-medicalise daunting EoL conversations.

## 3. Conclusions

Population ageing will result in an unprecedented surge in frail older persons with complex care needs that render untenable the current fragmented, facility-centric, doctor-based, and illness-centered healthcare system. To meet this challenge, the importance of healthy ageing was underlined in the public health framework for action in the recent World Report on Ageing and Health, which emphasises the pre-eminence of function and the importance of promoting intrinsic capacity beyond the focus on disease [5,6]. This raises the clarion call for a sustainable and evidence-based policy response for the systemic reform of healthcare delivery that is integrated, patient-centric, team-based, and health-centered through building partnerships and relationships that engage, empower, and activate patients and their support systems. Redesigning a responsive health system requires a top-down approach with financial incentives to service providers, the development of information systems to collect data for monitoring, and training a “frailty-ready” health and social care workforce [16]. To achieve long-term sustainability of the healthcare delivery system, partnerships will need to extend beyond the traditional healthcare–eldercare relationship to encompass broader whole-of-government and inter-agency collaboration, including the transport, housing, education, employment, and other related industries. Technology, data, design, and systems thinking will need to be factored into the planning of such services.

In this paper, we describe how the National Health Group and Alexandra Health System in the central region of Singapore leveraged upon a national reform of care delivery into region health clusters to develop initiatives to forge a frailty-ready healthcare system across the spectrum, including the well healthy (“living well”), well unhealthy (“living with illness”), unwell unhealthy (“living with frailty”), and the end-of-life (“dying well”). Whilst the early results are promising, data is needed to ascertain whether these initiatives translate into benefits in medium and long-term outcomes at the system and population levels. We are also mindful that there will be heterogeneity between different countries in designing healthcare systems that meet the WHO healthy ageing framework. Individual countries will need to consider the unique context of their healthcare system, including their particular needs as well as accompanying facilitators and barriers, to redesign healthcare delivery to meet the needs of the frail older population [16]. 

As pointed out in the WHO report, it is also critical to look into training and research [6]. To further advance the agenda of building a responsive frailty-ready healthcare system, it is important to ensure funding to build up capability in research and programme evaluation and conduct research with, not just for, older people founded on the principles of participation, collaboration, and action through evidence-balanced medicine [21,56]. It is also critical to ensure that there are resources to build and maintain a sustainable and appropriately trained workforce that includes healthcare professions and caregivers. To be “frailty-ready”, care providers should be equipped with basic gerontological and geriatric skills, as well as the more general competencies needed to work in integrated team-based systems, including communication, teamwork, and relevant information and communication technologies [57,58]. 

## Abbreviation

ACP	Advance care planning
AIP-CCT	Ageing-in-place community care teams
ComSA	Community for Successful Ageing
DFC	Dementia Friendly Communities
ED	Emergency department
EoL	End of life
FIFE	Framework for Inpatient care of the Frail Elderly
GFC	Geriatric Fracture Centre
KTPH	Khoo Teck Puat Hospital
NHG	National Healthcare Group
PWD	Persons with dementia
RHS	Regional Healthcare System
TC	Transitional Care
TTSH	Tan Tock Seng Hospital
WHO	World Health Organization

## References

[1] United Nations, Department of Economic and Social Affairs, Population Division (2017). World Population Prospects: The 2017 Revision, Key Findings and Advance Tables. https://esa.un.org/unpd/wpp/Publications/Files/WPP2017_KeyFindings.pdf.

[2] Dobriansky P.J., Suzman R.M., Hodes R.J. Why Population Aging Matters: A Global Perspective. https://www.nia.nih.gov/sites/default/files/2017-06/WPAM.pdf.

[3] GBD 2015 DALYs, HALE Collaborators (2016). Global, regional, and national disability-adjusted life-years (DALYs) for 315 diseases and injuries and healthy life expectancy (HALE), 1990–2015: A systematic analysis for the Global Burden of Disease Study 2015. Lancet.

[4] Fries J.F. (1980). Aging, Natural Death, and the Compression of Morbidity. N. Engl. J. Med..

[5] Beard J.R., Officer A., de Carvalho I.A., Sadana R., Pot A.M., Michel J.P., Lloyd-Sherlock P., Epping-Jordan J.E., Peeters G.M., Mahanani W.R. (2016). The World report on ageing and health: A policy framework for healthy ageing. Lancet.

[6] World Health Organization World Report on Ageing and Health. http://www.who.int/ageing/publications/world-report-2015/en/.

[7] Woo J., Leung J., Zhang T. (2016). Successful Aging and Frailty: Opposite Sides of the Same Coin?. J. Am. Med. Dir. Assoc..

[8] Clegg A., Young J., Iliffe S., Rikkert M.O., Rockwood K. (2013). Frailty in elderly people. Lancet.

[9] Dent E., Lien C., Lim W.S., Wong W.C., Wong C.H., Ng T.P., Woo J., Dong B., de la Vega S., Poi P.J.H. (2017). The Asia-Pacific clinical practice guidelines for the management of frailty. J. Am. Med. Dir. Assoc..

[10] Cesari M., Prince M., Thiyagarajan J.A., De Carvalho I.A., Bernabei R., Chan P., Gutierrez-Robledo L.M., Michel J.P., Morley J.E., Ong P. (2017). Frailty: An emerging public health priority. J. Am. Med. Dir. Assoc..

[11] Morley J.E., Vellas B., Van Kan G.A., Anker S.D., Bauer J.M., Bernabei R., Cesari M., Chumlea W.C., Doehner W., Evans J. (2013). Frailty consensus: A call to action. J. Am. Med. Dir. Assoc..

[12] Sirven N., Rapp T. (2017). The cost of frailty in France. Eur. J. Health Econ..

[13] Lim W.S., Cheah W.K., Ali N., Han H.C., Anthony P.V., Chan M., Chong M.S. (2014). Worry about performance: A unique dimension of caregiver burden. Int. Psychogeriatr..

[14] Chong M.S., Tan W.S., Chan M., Lim W.S., Ali N., Ang Y.Y., Chua K.C. (2014). Cost of informal care for community-dwelling mild-moderate dementia patients in a developed Southeast Asian country. Int. Psychogeriatr..

[15] Upshur R.E., Tracy S. (2008). Chronicity and complexity: Is what’s good for the diseases always good for the patients?. Can. Fam. Physician.

[16] Woo J. (2017). Designing Fit for Purpose Health and Social Services for Ageing Populations. Int. J. Environ. Res. Public Health.

[17] Singapore Department of Statistics Population and Population Structure. http://www.singstat.gov.sg/statistics/browse-by-theme/population-and-population-structure.

[18] Gan K.Y. Geriatrics beyond Borders: Are We Frailty Ready?. https://www.moh.gov.sg/content/moh_web/home/pressRoom/speeches_d/2016/speech-by-mr-gan-kim-yong--minister-of-health--at-the-asia-pacif.html.

[19] Cheah J. (2001). Chronic disease management: A Singapore perspective. BMJ.

[20] Choo P.W.J. Forging a frailty-ready healthcare system: From illness-care to health-care. Proceedings of the Asia Pacific Geriatric Conference.

[21] Lim W.S., Ding Y.Y. (2015). Evidence-balanced medicine: “Real” evidence-based medicine in the elderly. Ann Acad. Med. Singap..

[22] Chong E., Ho E., Baldevarona-Llego J., Chan M., Wu L., Tay L. (2017). Frailty and Risk of Adverse Outcomes in Hospitalized Older Adults: A Comparison of Different Frailty Measures. J. Am. Med. Dir. Assoc..

[23] (2012). Healthcare 2020: Improving Accessibility, Quality & Affordability. https://www.moh.gov.sg/content/dam/moh_web/healthscope/archive/2012/MOH%20Healthscope_July-August%202012%20Issue.pdf.

[24] Gan K.Y. MOH Committee of Supply Debate 2016. https://www.moh.gov.sg/content/moh_web/home/pressRoom/speeches_d/2016/speech-by-minister-fo-health--mr-gan-kim-yong--at-the-moh-commit.html.

[25] Gan K.Y. Reorganisation of Healthcare System into Three Integrated Clusters to Better Meet Future Healthcare Needs. https://www.moh.gov.sg/content/moh_web/home/pressRoom/pressRoomItemRelease/2017/reorganisation-of-healthcare-system-into-three-integrated-cluste.html.

[26] Hibbard J.H., Greene J. (2013). What the evidence shows about patient activation: Better health outcomes and care experiences; fewer data on costs. Health Affairs.

[27] Gan K.Y. Futurescape: Home Healthcare—Local and International Perspectives. https://www.moh.gov.sg/content/moh_web/home/pressRoom/speeches_d/2016/speech-by-mr-gan-kim-yong--minister-for-health--at-home-nursing-.html.

[28] Teo N., Gao Q., Nyunt M.S.Z., Wee S.L., Ng T.P. (2017). Social Frailty and Functional Disability: Findings from the Singapore Longitudinal Ageing Studies. J. Am. Med. Dir. Assoc..

[29] Kiyota E., Tanaka Y., Arnold M., Aldrich D.P. Elders Leading the Way to Resilience. https://papers.ssrn.com/sol3/papers.cfm?abstract_id=2575382.

[30] Wong S.F. Building Health Systems to Support Physical Activity & Active Ageing. Proceedings of the Asia Pacific Geriatric Conference.

[31] Forget Us Not. Dementia Friendly Community. https://forgetusnot.sg/dementia-friendly-community.html.

[32] Steels S. (2015). Key characteristics of age-friendly cities and communities: A review. Cities.

[33] (2017). 24-Hour Dementia Go-to Point Opens in Yishun. http://www.straitstimes.com/singapore/24-hour-dementia-go-to-point-opens-in-yishun.

[34] Gan K.Y. Bed Crunch. https://www.moh.gov.sg/content/moh_web/home/pressRoom/Parliamentary_QA/2014/bed-crunch.html.

[35] Wong S.K.Y. (2014). Advanced Planning Eases End-Stage Suffering. https://www.ttsh.com.sg/page.aspx?id=6076.

[36] Salvi F., Morichi V., Grilli A., Giorgi R., De Tommaso G., Dessì-Fulgher P. (2007). The elderly in the emergency department: A critical review of problems and solutions. Intern. Emerg. Med..

[37] Foo C.L., Siu V.W.Y., Tan T.L., Ding Y.Y., Seow E. (2011). Geriatric assessment and intervention in an emergency department observation unit reduced re-attendance and hospitalization rates. Australas. J. Ageing.

[38] Foo C.L., Siu V.W.Y., Ang H., Phuah M.W.L., Ooi C.K. (2014). Risk stratification and rapid geriatric screening in an emergency department—A quasi-randomized controlled trial. BMC Geriatr..

[39] Teh C.R., Lim W.S., Basri R., Ismail N.H. (2006). Utility of a patient-response screening question for visual impairment. J. Am. Geriatr. Soc..

[40] Landefeld C.S., Palmer R.M., Kresevic D.M., Fortinsky R.H., Kowal J. (1995). A randomized trial of care in a hospital medical unit especially designed to improve the functional outcomes of acutely ill older patients. N. Engl. J. Med..

[41] Fulmer T. (2001). The Geriatric Resource Nurse: A model of caring for older patients. Am. J. Nurs..

[42] Inouye S.K., Bogardus S.T., Charpentier P.A., Leo-Summers L., Acampora D., Holford T.R., Cooney L.M. (1999). A multicomponent intervention to prevent delirium in hospitalized older patients. N. Engl. J. Med..

[43] Friedman S.M., Mendelson D.A., Bingham K.W., Kates S.L. (2009). Impact of a comanaged geriatric fracture centre on short-term hip fracture outcomes. Arch. Intern. Med..

[44] Ramason R., Chong M.S., Chan W., Rajamoney G.N. (2014). Innovations in hip fracture care: A comparison of geriatric fracture centers. J. Am. Med. Dir. Assoc..

[45] Tan K.Y., Tan P., Tan L. (2011). A collaborative transdisciplinary “geriatric surgery service” ensures consistent successful outcomes in elderly colorectal surgery patients. World J. Surg..

[46] Tan K.Y., Kawamura Y.J., Tokomitsu A., Tang T. (2012). Assessment for frailty is useful for predicting morbidity in elderly patients undergoing colorectal cancer resection whose comorbidities are already optimized. Am. J. Surg..

[47] Chia C.L., Mantoo S.K., Tan K.Y. (2016). ‘Start to finish trans-institutional transdisciplinary care’: A novel approach improves colorectal surgical results in frail elderly patients. Colorectal Dis..

[48] Wang Z., Tan K.Y., Tan P. (2013). Functional outcomes in elderly adults who have undergone major colorectal surgery. J. Am. Geriatr. Soc..

[49] Alexandra Health System’s Ageing-in-Place Programme—First Singapore Public Healthcare Programme to Win 2014 UN Public Service Award. https://www.ktph.com.sg/uploads/1403773586Media%20Release%20-%20First%20Singapore%20Public%20Healthcare%20Programme%20to%20Win%202014%20UN%20.

[50] Community for Successful Ageing (ComSA). https://tsaofoundation.org/what-we-do/comsa/about-comsa.

[51] Report on the National Strategy for Palliative Care. https://www.duke-nus.edu.sg/sites/default/files/Report_on_National_Strategy_for_Palliative_Care%205Jan2012.pdf.

[52] National Guidelines for Palliative Care. https://singaporehospice.org.sg/shc/wp-content/uploads/2016/09/NGPC2015Jan20.pdf.

[53] Teno J.M., Gruneir A., Schwartz Z., Nanda A., Wetle T. (2007). Association between advance directives and quality of end-of-life care: A national study. J. Am. Geriatr. Soc..

[54] Detering K.M., Hancock A.D., Reade M.C., Silvester W. (2010). The impact of advance care planning on end of life care in elderly patients: Randomised controlled trial. BMJ.

[55] Living Matters. https://www.livingmatters.sg/.

[56] Ch’ng A.S.H., Yong K.T.W., Ng D.W.H., Heyzer L., Lim W.S. (2017). Trends in Geriatrics-Related Translational Research and Funding in the “Big 4” Medical Journals. J. Am. Med. Dir. Assoc..

[57] Lee S.Y., Dong L., Lim Y.H., Poh C.L., Lim W.S. (2016). SBAR: Towards a common interprofessional team-based communication tool. Med. Educ..

[58] Tan K.T., Adzhahar F.B., Lim I., Chan M., Lim W.S. (2014). Transactive memory system as a measure of collaborative practice in a geriatrics team: Implications for continuing interprofessional education. J. Interprof. Care.

